# Eye movements do not preferentially test inferences in the blind spot

**DOI:** 10.1038/s41598-025-31647-1

**Published:** 2025-12-10

**Authors:** Cemre Baykan, Alexander C. Schütz

**Affiliations:** AG Sensomotorisches Lernen, Fachbereich Psychologie, Philipps-Universität Marburg, Marburg, Hessen Germany

**Keywords:** Perceptual completion, Filling-in, Blind spot, Saccade, Eye movement, Neuroscience, Psychology, Psychology

## Abstract

Humans make eye movements to regions of high uncertainty to maximize the information gain in their visual search. Along with veridical sensory information, there is also perceptually inferred information that arises at the gaps caused by anatomical or environmental factors. It is unclear how those inferences are treated in comparison to veridical information during search behavior. Here, in two experiments, we tested if eye movements are preferentially directed towards the blind spot as an area of high uncertainty and high information gain in a monocular visual search task. The results show that the first saccade was not directed primarily to the blind spot when “invisible” targets in the left and right blind spot occured interleaved. Only when viewing conditions were blocked, such that “invisible” targets occured always in the same blind spot side, participants learned to look preferentially at the blind spot. These results show that perceptual inferences in the blind spot are not preferentially tested by eye movements in general, but that they can be optimized by using contextual information.

## Introduction

 Eye movements can be considered as experiments to test perceptual inferences^1^. When identifying objects or making decisions about their properties, humans make saccadic eye movements to project the target object on the center of the visual field, the fovea. This is because foveal vision has higher spatial resolution, visual clarity and contrast sensitivity than the periphery^2–6^. Saccadic eye movements can be driven by both low-level stimulus features and high-level mechanisms^7,8^ such as stimulus salience^9^, objects and faces^10,11^, execution of an active or planned task^12,13^ and reward^14^. It has been found that eye movements can optimize visual search by targeting regions of high uncertainty^15,16^. In these studies, observers searched for a Gabor target that was presented in a random pink noise background. When the statistics of their saccades were compared with those of an ideal Bayesian observer that uses knowledge of the visibility map, the results showed a close match between human and ideal human search behavior. This finding suggests that human search behavior can be close to a search strategy that maximizes information gain. However, there are also other scenarios, where eye movements clearly are not optimal. In the case of multiple-target search, it was found that human search behavior does not follow a strategy that maximizes information gain: observers made saccades towards the most likely target locations rather than targeting rare or more uncertain target locations^17,18^. There are also circumstances in which observers ignore probability information about target location and make a saccade to a location that is closer to their current fixation point^19^. Moreover, studies showed that visual search depends on spatial layouts of a visual scene^20,21^ and does not necessarily incorporate perceptual uncertainty in the control of eye movements^22^.

While perceptual uncertainty can be estimated based on the visibility of a search target at different locations in the visual field, it is objectively maximal in gaps of sensory information. Such gaps can occur because of properties of the own sensory system, such as natural or pathological scotomata or because of properties of the environment, such as occlusion^23,24^. The blind spot is a natural scotoma that is caused by the absence of photoreceptors in the region where the optic nerve leaves the eyeball^24–26^. Despite these spatial gaps in the available sensory information, we have a seamless perceptual experience. Our brain compensates or fills-in this missing information based on surrounding details or input from the other eye^27–29^. Perceptual filling-in at the blind spot occurs almost instantaneously and outside of individuals’ awareness^30,31^. Yet, previous research suggests that filled-in (inferred) information across the blind spot differs from sensory information in various ways: on the one hand, perceptually filled-in stimuli have weaker strength during binocular rivalry^32^, and evoke weaker brain responses^33^, on the other hand individuals respond to those stimuli with faster reaction time and overconfidence^34^. In addition to these differences, it is an open question how inferred percepts are used in the control of eye movements.

When we encounter ambiguous sensory information, the nervous system makes a perceptual decision that is more likely or more reliable depending on prior experience or context^35^. Interestingly, however, when individuals are confronted with both veridical and inferred percepts simultaneously, they tend to show a bias for inferred percepts that are objectively less reliable^34,36^. For instance, Ehinger et al.^34^ found that observers choose a stimulus presented inside the blind spot more as continuous than one presented outside the blind spot, implying their trust for an inferred percept over veridical information. In another study, Gloriani and Schütz^36^ asked participants to discriminate between continuous and discontinuous stimuli in the fovea and the periphery and to select which stimulus they prefer to judge in a confidence task. They found that individuals prefer a foveal stimulus more often than a peripheral one under both photopic (light-adapted) and scotopic (dark-adapted) viewing conditions although there is a foveal scotoma under scotopic viewing. Altogether, these studies provide some examples in which individuals show a perceptual bias, in their confidence judgments, for perceptually filled-in information. It is still not known if observers will demonstrate a motor bias, in the control of eye movements, for these stimuli.

Commonalities and dissociations between perception and eye movements have been investigated in many studies^37–39^ and dissociations have been observed for different types of eye movements and different experimental paradigms^40–42^. Hence, it is possible that eye movements treat the uncertainty of the inferred information in the blind spot differently than conscious perception and metacognition.

Here, we investigated if perceptual inferences in the blind spot, as an area of high uncertainty and high information gain, are tested preferentially by eye movements in visual search. We used a monocular search paradigm in which participants viewed a search display through a stereoscopic device that allows for independent stimulation of the right and the left eye. The search patches were presented in three viewing conditions either to both eyes in binocular viewing or to only one eye in monocular viewing. Depending on the side of the target (right or left), it could fall on the blind spot in monocular viewing. When the target fell inside the blind spot, it was initially “invisible” to participants and therefore its location was uncertain. We manipulated the predictability of “invisible” target locations by randomizing viewing conditions (Experiment 1) or by presenting them in separate blocks (Experiment 2), such that the target was always visible when presented on one side and always “invisible” (i.e. in the blind spot) when presented on the other side. We explored two possibilities explaining the search behavior for a target that was located either at the blind spot or an intact region. If the inferred information in the blind spot is not considered as reliable, observers may look primarily at the blind spot location to reduce the uncertainty and to maximize the information gain. Conversely, if inferred information in the blind spot is treated as more reliable information than sensory information elsewhere, observers may make a saccade to the intact region.

## Methods

### Participants

37 and 35 participants with either normal or corrected-to-normal visual acuity took part in Experiment 1 and 2, respectively. We inspected participants’ accuracy by examining the behavioral performance and eye movement data in the binocular viewing condition. In the binocular viewing, whenever the target was presented peripherally, we expected participants to see the target reliably. If a participant performed the joint task, which was both to look at the correct target side and to give the correct answer, in less than 60% of all trials correctly, we excluded them from further analyses (6 and 5 participants in Experiment 1 and 2, respectively). Additionally, we excluded one participant in Experiment 2 due to high saccade latency. As a result, we entered the data of 31 participants (26 females, 5 males, average age 21.2 years, age range: 18–37) to the main analyses in Experiment 1, and 29 participants (17 females, 12 males, average age 27.3 years, age range: 19–46) in Experiment 2.

All participants were naive to the purpose of the study and gave written informed consent before the experiments started. Each participant was compensated by a monetary payment of 8 euros per hour or a course credit in Experiment 1 and 10 euros per hour in Experiment 2 for their participation. The experiments were approved by the ethics committee of the Psychology Department at the University of Marburg (proposal number 2021-71k) and carried out in accordance with the relevant guidelines and regulations and the Declaration of Helsinki.

### Apparatus and stimuli

The experimental routine and stimuli were generated in MATLAB (Version R2023a; Mathworks, Inc.) using the Psychophysics Toolbox^43^. For the stimulus presentation, we used a mirror stereoscope setup, where a mirror was placed in front of each eye at ± 45 degrees to the line of sight. In this way, images from two computer displays (resolution 1920 × 1080, height 29.9 cm, width 53.1 cm, refresh rate 120 Hz) located to the left and to the right from the participant were directed to each eye separately. The mirrors were cold mirrors (Edmund Optics Ltd, York, UK), which are reflecting the visible spectrum of the light but are transparent for infra-red light, so that the infra-red eye tracker could record eye movements through the mirrors^44^. Participants were seated at a viewing distance of 73 cm fixed with a chin and forehead rest. The experiment was conducted in a dimly-lit and sound-attenuated experimental room.

**Fig. 1:**
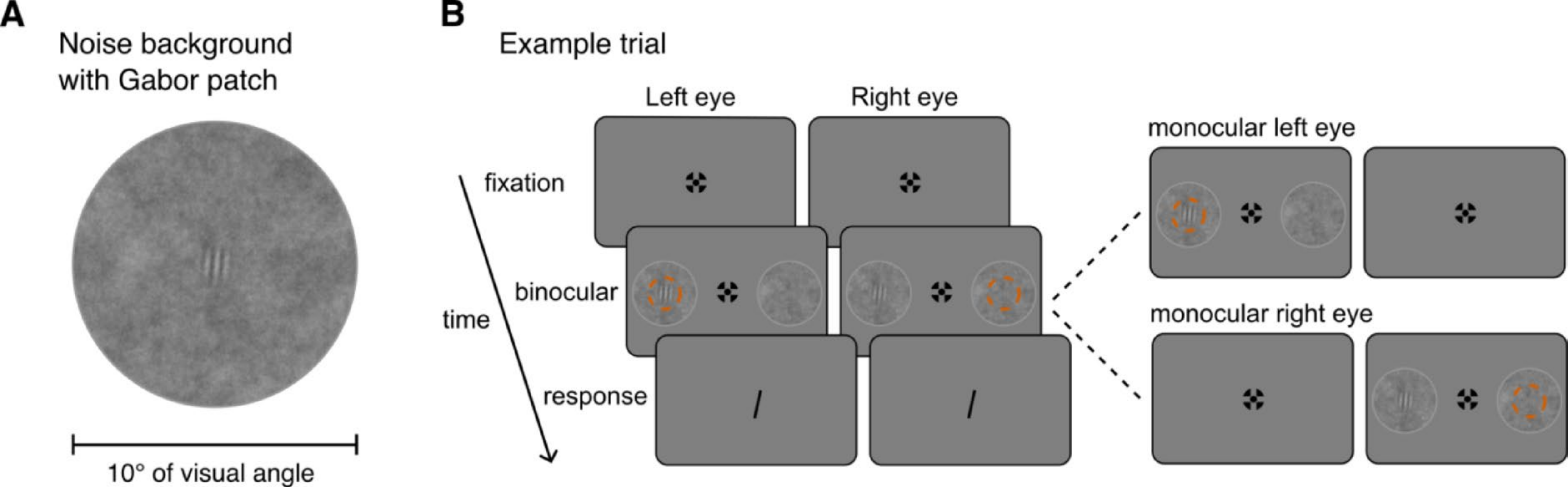
**A** The search target (a Gabor patch) on a noise background. **B** Schematic trial sequence used in two experiments: each trial started with a fixation cross presented until a key press; next, the search stimuli were presented - either binocularly, or monocularly to the left or the right eye in different trials; participants had to find a small, striped target inside either of the two noise patches (in this example on the left), which were presented left and right from initial fixation; stimulus presentation was followed by a response screen where the participant could report the target’s orientation. The orange dashed circle was not part of the display and indicates the location of the blind spot of the left and right eye, where the target would be invisible during central fixation. Stimuli are not drawn to scale.

Figure 1A illustrates the stimuli used in both experiments, the noise background with a search target. All stimuli were presented on a gray (luminance of 64.85 cd/m^2^, RGB values of 128, 128, 128) background. The search target was a Gabor patch with a spatial frequency of 3 cycles/° and a contrast of 0.3 root-mean-square (rms), tilted 5° to the right or to the left. The size of the search target (diameter of 2°) was chosen so that it could not be seen when presented in the blind spot, considering its size of about 6° x 5°^45^ and its non-sharp borders^46,47^. The target was presented on a pink (1/f) noise background (diameter 10° of visual angle, rms contrast 0.07), as it has been suggested to have the same spatial power spectra as natural scene images^48^. The noise background was encircled with a 0.09°-wide gray circle (luminance of 75.84 cd/m^2^, RGB values of 150, 150, 150). The fixation target shape (outer diameter 0.6°, inner diameter 0.15°) was a combination of a bulls eye and a cross hair, as it was reported to result in the best stability of fixation^49^. Along with the fixation target, we presented two noise background stimuli that were away from the center of the monitor by 14° horizontally and 1.5° vertically below, targeting the blind spot location^50,51^.

### Procedure and design

Before the main experiments, participants performed a stereo vision test. In each trial, we presented four white circles (luminance of 128.29 cd/m^2^, diameter 1.07°) that were placed away from the center of the monitor by 1.5°. There was a 0.43°-wide gray ring (luminance of 75.84 cd/m^2^, RGB values of 150, 150, 150, diameter 9.43°) around these four target circles. There was also a fixation target at the center of the monitor (outer diameter 0.6°, inner diameter 0.15°). The fixation target and the gray ring were presented to both eyes to help participants to fuse the information that came from two eyes. In the stereo vision test, there were 24 trials, 12 with crossed disparity and 12 with uncrossed disparity. In the crossed disparity trials, the stimulus was shifted rightwards on the left eye and leftwards on the right eye, forming a ‘closer’ perception of the stimulus. In the uncrossed disparity trials, the stimulus was shifted leftwards on the left eye and rightwards on the right eye, forming a ‘farther’ perception. The shift was either 0.15° or 0.3° (equal amount of trials). A randomly chosen circle was selected as an oddball having the crossed disparity while the other three circles having the uncrossed disparity in half of the trials. In the other half of the trials, the oddball had the uncrossed disparity and the other three circles had the crossed disparity. Participants were asked to report if the oddball looked closer or further away.

In Experiment 1, following the stereo vision test, participants had 9 trials of practice. There were 3 viewing conditions (binocular, monocular left, monocular right) and 3 target side conditions (left, right, none). In binocular viewing, participants could see the Gabor target when it was presented either on the left or right side. In monocular viewing, participants could not see the target when it fell into the blind spot: monocular left eye viewing with the left side target and monocular right eye viewing with the right side target. When the target was presented on the other side, they could see the target. In the main experiment, each condition was repeated 40 times, in half of those, the Gabor pattern had a 5°-tilt to the left and in the other half a 5°-tilt to the right relative to the vertical. In total, there were 360 trials, randomly presented in 3 blocks. Each of these conditions was presented once in the practice session. A trial started when participants fixated on the fixation cross and pressed a button. In each trial, two noise backgrounds with Gabor patch were presented on either side of the fixation cross (see Fig. 1B). When participants fixated at the central fixation cross, we expected participants to recognize the target location (right or left). Yet, if participants make a saccade towards the target, their orientation discrimination performance would increase. Therefore, we instructed participants to first make a saccade to the target side and then report its orientation. Once they made a horizontal saccade > 8° from the fixation cross, the stimulus display was terminated after 200 ms. A probe line that was tilted from vertical, 5° to the right or to the left, randomly, was shown on the response screen. Participants could swap the tilt direction using a button press and register their response using another button.

In Experiment 2, following the stereo vision test, participants started the main experiment. There were 3 viewing conditions (binocular, monocular left, monocular right) and 2 target side conditions (left, right). Each condition was repeated 40 times, in half of those, the Gabor pattern had a 5°-tilt to the left and in the other half a 5°-tilt to the right relative to the vertical. In total, there were 240 trials, each viewing condition presented block-wise. All participants started with a binocular viewing condition block, after which the order of the blocks was randomized and counterbalanced among participants. The trial sequence was the same as in Experiment 1 (see Fig. 1B).

### Eye movement recording and analysis

We performed eye tracking using the Eyelink 1000 system (SR Research, Ontario, Canada). A 9-point calibration procedure was performed at the beginning of the experiment. Each trial started with an automatic drift check. In Experiment 2, we performed additional calibration procedures before each block.

For each participant and trial, we used a custom function to categorise if participants looked to the right- or the left-target. The function examined the first large stimulus-driven saccade (more than 5.4°) to one direction and categorised it as that position.

### Statistical analyses

All data analyses were performed in R (version 4.3.2)^52^. We analyzed the behavioral and eye movement data using pairwise t-test via *stats* (version 4.3.2)^52^ package.

For each participant, we calculated the proportion of correct responses and correct saccades grouped by the three main conditions: when the target was presented in the blind spot (IN), outside of the blind spot (OUT) or binocularly (BINO). The IN condition consisted of the averaged responses of the two blind spot conditions (monocular right eye - right side target and monocular left eye - left side target), and the OUT condition consisted of the averaged responses of the non-blind spot conditions (monocular right eye - left side target and monocular left eye - right side target).

## Results

### Experiment 1

**Fig. 2:**
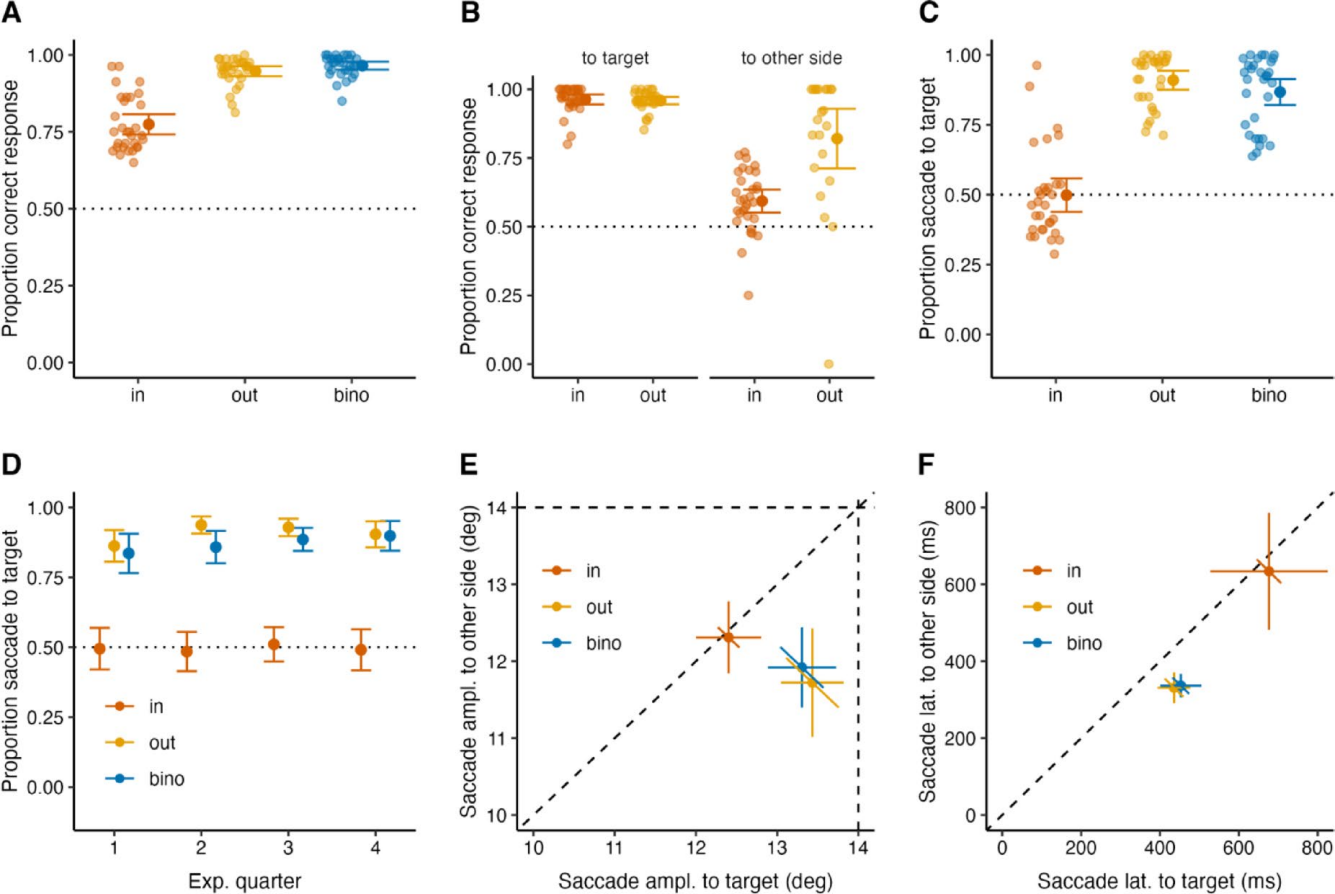
Perceptual and eye movement results of the first experiment with interleaved conditions. We report the results grouped by the target presentation condition: IN, in the blind spots (orange); OUT, outside of the blind spots (yellow); BINO, binocular viewing (blue). Horizontal dotted lines indicate 50% chance level in (A - D). Error bars are 95% confidence intervals. **A** Proportion of correct responses in the orientation discrimination task. **B** Proportion of correct responses depending on whether the target was presented inside or outside of the blind spot and whether the saccade was made to the target or the other side. **C** Proportion of saccades to the target side. **D** Proportion of saccades to the target side for quarters across the whole experiment. **E** Saccade amplitudes to possible target locations: to target, x-axis; to other side, y-axis. Diagonal dashed line shows equal saccade amplitude. Horizontal and vertical dashed lines represent the target eccentricity. **F** Saccade latencies to possible target locations: to target, x-axis; to other side, y-axis. Diagonal dashed line shows equal saccade latency. Horizontal and vertical error bars in (E) and (F): confidence intervals; diagonal error bars: confidence intervals for pairwise differences (x-y)^53^.

We asked participants to search for a small target that was presented in either of two large horizontally displaced noise patches and to report its orientation (Fig. 1B). In the first experiment, we had control trials for each of the three viewing conditions (binocular, monocular left eye, monocular right eye) in which no target was present. To understand if participants had a side bias while making a saccade, we examined the binocular viewing condition when no target was present: the mean proportion gaze to the left side (0.50 ± 0.03), *t*(30) = −0.14, *p* =.89, *d* = 0.03, was not different from chance level of 0.5, indicating no bias for a side.

Figure 2 shows the results of the perceptual task and the eye movements in the first experiment. First, we assessed the reliability of participants’ performance by examining their perceptual performance in the BINO and OUT conditions. Figure 2 A shows the proportion of correct responses in the orientation discrimination task. Participants responded to the target orientation with high accuracy in both conditions: their proportion of correct responses (± SEM) was 0.97 (± 0.01) for the BINO and 0.95 (± 0.01) the OUT conditions. This was quite different for the IN condition: the proportion of correct responses were 0.77 (± 0.02), and it was lower than for OUT (t(30) = −11.28, *p* <.0001, d = −2.02) and for BINO (t(30) = −11.49, *p* <.0001, d = −2.06).

Next, we examined if participants missed information by not making a saccade to the blind spot side. To test this, we calculated the proportion of correct orientation responses of IN and OUT conditions, separately for trials with saccades to the target or the other side. Figure 2B shows the proportion of correct responses depending on saccade direction when the target was presented inside (IN) or outside (OUT) of the blind spots. When participants made a saccade to the target side, their orientation discrimination accuracy was high: the proportion of correct responses was 0.96 (± 0.01) and 0.96 (± 0.01) for the OUT and IN conditions, respectively. However, when participants made a saccade to the other side, their orientation discrimination accuracy dropped: the proportion of correct responses was 0.82 (± 0.05) for the OUT and 0.59 (± 0.02) for the IN condition. These results suggest that observers missed information when not gazing at the blind spot side.

Figure 2 C shows the proportion of correct saccades to the target side in all viewing conditions. We tested if participants preferentially looked at the blind spot region by examining the proportion of saccades to the target side. Participants showed no preference for the blind spot: their proportion of saccades to the target side for the IN condition (mean ± SEM, 0.50 ± 0.03) did not differ from 0.5 chance level (t(30) = −0.06, *p* =.96, d = 0.01) and it was lower than for OUT (t(30) = −13.65, *p* <.0001, d = −2.45) and for BINO (t(30) = −11.48, *p* <.0001, d = −2.06). This result indicates that participants did not preferentially saccade to the blind spot side when they did not see the target. We also examined whether participants’ saccade preference improved over trials. We averaged the proportion of saccades to the target side in four bins of trials across the whole experiment and fitted a linear regression to each condition to estimate the development of performance. We compared the slope values of each condition against zero using one sample *t*-test. The proportion of saccades to the target side did not increase for the IN (t(30) = 0.17, *p* =.87, d = 0.03) and OUT (t(30) = 1.47, *p* =.15, d = 0.26) conditions, but there was a positive slope for the BINO condition (t(30) = 2.16, *p* =.04, d = 0.39) (Fig. 2D).

Figure 2E shows the saccade amplitudes to possible target locations. When participants made a saccade to the target side, their saccade amplitude nearly corresponded to the target location (14°) for BINO and OUT conditions: 13.3° (± 0.2) and 13.4° (± 0.2), respectively, and it significantly decreased to 11.9° (± 0.3) for the BINO (t(25) = 5.35, *p* <.001, d = 1.18) and to 11.7° (± 0.3) for the OUT (t(23) = 5.47, *p* <.001, d = 1.27) condition when they made a saccade to the other side. Although smaller, these saccades went well beyond the inner edge of the noise (9°) and brought the gaze inside the area of the noise. Participants’ saccade amplitudes were comparable for the IN condition when they made a saccade to the target side (12.4° ± 0.2) and to the other side (12.3° ± 0.2), t(30) = 0.72, *p* =.48, d = 0.08. These results show that saccade amplitudes were larger and more accurate when the saccade was correctly directed at a visible target in the BINO and OUT conditions. Saccades to an “invisible” target in the IN condition or to the other side without a target were less accurate.

Figure 2F shows the saccade latencies to possible target locations. Participants’ saccade latencies were comparable for the IN condition when they made a saccade to the target side (676.5 ± 73 ms) and to the other side (634 ± 75 ms), t(30) = 1.40, *p* =.17, d = 0.10. Meanwhile, participants’ saccade latencies were longer when they made a saccade to the target side in comparison to the other side for the BINO, 452.9 ± 25.5 versus 336.5 ± 14.8 (t(25) = 5.69, *p* <.001, d = 1.1), and for the OUT, 435.6 ± 20.1 versus 331 ± 19.4 (t(23) = 4.57, *p* =.0001, d = 1.08) condition. This suggests that “incorrect” saccades to the opposite side of a visible target might have been triggered prematurely, reflecting a speed-accuracy trade-off^54,55^, or the influence of predictive signals, such as repetition priming^56–59^. Participants required more time to saccade in either direction whenever the target was invisible in the blind spot, which could reflect a transition from reactive, stimulus-driven saccades to more voluntary, exploration-driven saccades.

### Experiment 2

**Fig. 3:**
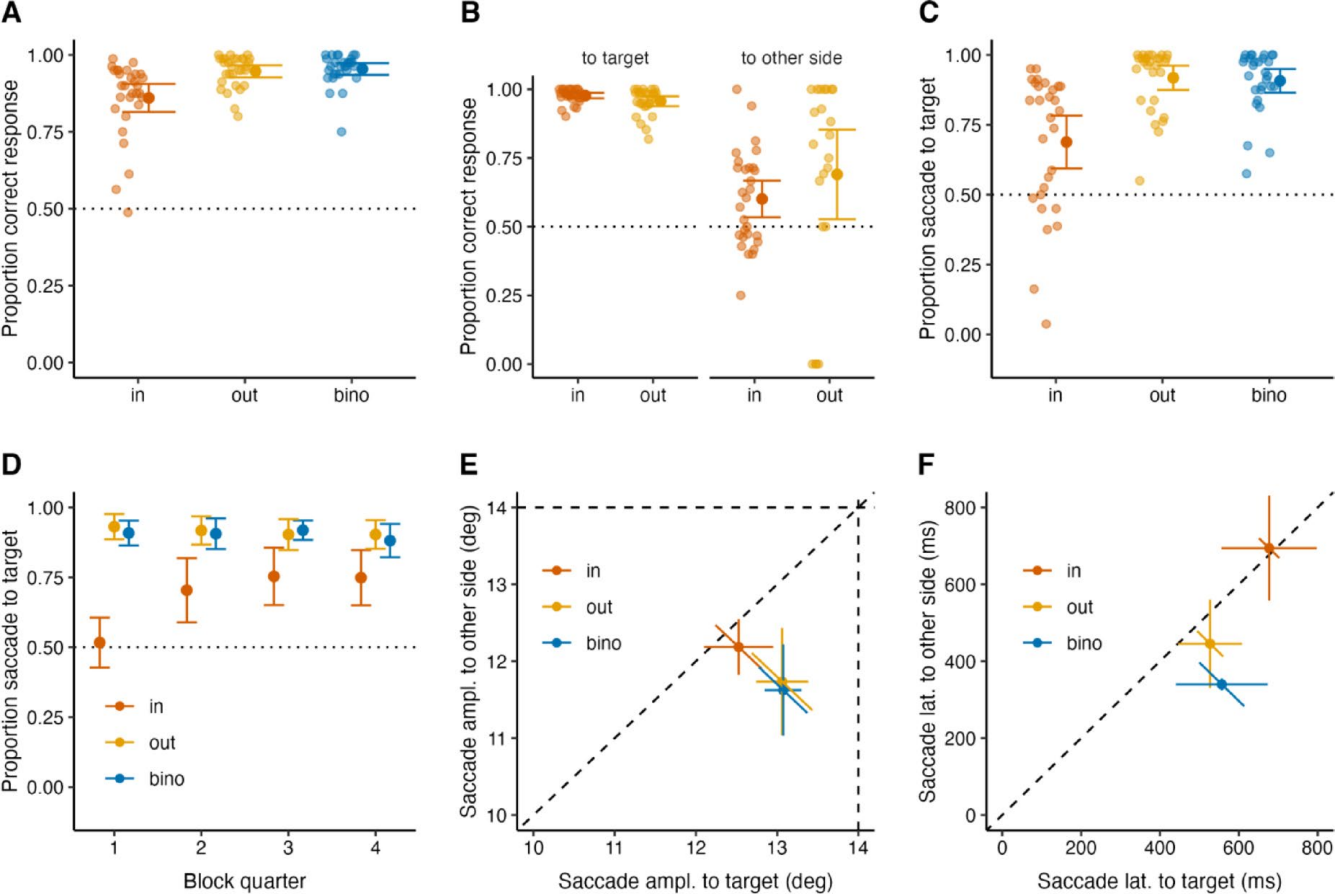
Perceptual and eye movement results of the second experiment with blocked viewing conditions. Conventions are the same as in Fig. 2. We report the results grouped by the target presentation condition: IN, in the blind spots (orange); OUT, outside of the blind spots (yellow); BINO, binocular viewing (blue). Horizontal dotted lines indicate 50% chance level in (A–D). Error bars are 95% confidence intervals. ***A*** Proportion of correct responses in the orientation discrimination task. ***B*** Proportion of correct responses depending on whether the target was presented inside or outside of the blind spots and whether the participants made a saccade to the target or the other side. ***C*** Proportion of saccades to the target side. ***D*** Proportion of saccades to the target side for quarters per block of Experiment 2. ***E*** Saccade amplitudes to possible target locations: to target, x-axis; to other side, y-axis. Diagonal dashed line shows equal saccade amplitude. Horizontal and vertical dashed lines represent the target eccentricity. ***F*** Saccade latencies to possible target locations: to target, x-axis; to other side, y-axis. Diagonal dashed line shows equal saccade latency. Horizontal and vertical error bars in (E) and (F): confidence intervals; diagonal error bars: confidence intervals for pairwise differences (x-y)^53^.

In the second experiment, the viewing conditions (binocular, monocular left eye, monocular right eye) were presented in a block-wise manner. Given that there was no side bias while making saccades towards a target in the first experiment, we did not include any trials without a target in the second experiment.

Figure 3 shows the perceptual and eye movement results of the second experiment. Figure 3 A shows the proportion of correct responses in the orientation discrimination task. Like in the first experiment, in Experiment 2, participants responded to the target orientation with high accuracy when they could see the target: their proportion correct response was 0.95 (± 0.01) for the BINO and 0.95 (± 0.01) for the OUT condition, indicating a reliable performance. Their proportion of correct responses decreased to 0.86 (± 0.02) for the IN condition, and it was lower than for OUT (t(28) = −3.89, *p* <.0001, d = −0.72) and for BINO (t(28) = −5.54, *p* <.0001, d = −1.03). Importantly, participants reached a performance that was higher than the one in Experiment 1 for the IN condition, t(50.85) = 2.96, *p* =.002, d = 0.77.

Figure 3B shows the proportion of correct responses depending on saccade direction when the target was presented inside (IN) or outside (OUT) of the blind spots. When participants made a saccade to the target side, their orientation discrimination accuracy was high: the proportion of correct responses was 0.96 (± 0.01) for the OUT and 0.98 (± 0.01) for the IN conditions. However, when participants made a saccade to the other side, their orientation discrimination accuracy dropped: the proportion of correct responses was 0.69 (± 0.08) for the OUT and 0.60 (± 0.03) for the IN conditions. These results suggest that observers missed information when not gazing at the target when it was presented in the blind spot.

Figure 3 C shows the proportion of saccades to the target side in all viewing conditions. The proportion of saccades to the target side was 0.91 (± 0.02) for the BINO, 0.92 (± 0.02) for the OUT, and 0.69 (± 0.05) for the IN condition which was higher than chance level (t(28) = 4.08, *p* =.0003, d = 0.76). Compared to the results of Experiment 1, this indicates that participants directed their first saccade to the blind spot only when the blind-spot side was presented in a block-wise manner and was predictable by contextual information. Remarkably, participants learned the “invisible” target location over a few trials and kept their performance stable throughout the rest of the experiment (Fig. 3D). The proportion of saccades to the target side increased over time for the IN condition (t(28) = 7.63, *p* <.0001, d = 1.42), while it did not increase for the OUT (t(28) = −1.61, *p* =.12, d = 0.30) and the BINO (t(28) = −0.99, *p* =.33, d = 0.18) condition.

Figure 3E shows the saccade amplitudes to possible target locations. When participants made a saccade to the target side, their saccade amplitude was close to the target location (14°) for the BINO and OUT conditions: 13.1° (± 0.1) and 13.1° (± 0.2), respectively, and it significantly decreased to 11.6° (± 0.3) for the BINO (t(21) = 5.08, *p* <.001, d = 1.43) and to 11.7° (± 0.3) for the OUT (t(21) = 3.7, *p* =.001, d = 1.08) condition when the saccade was made to the other side. Participants’ saccade amplitudes were comparable for the IN condition when they made a saccade to the target side (12.5° ± 0.2) and to the other side (12.2° ± 0.2), t(28) = 1.22, *p* =.23, d = 0.33.

Figure 3 F shows the saccade latencies to possible target locations. Participants’ saccade latencies were comparable for the IN condition when they made a saccade to the target side (676.9 ± 59 ms) and to the other side (694.1 ± 67 ms), t(28) = −0.69, *p* =.50, d = 0.05. Meanwhile, participants’ saccade latencies were longer when they made a saccade to the target side in comparison to the other side for the BINO, 556.8 ± 56 versus 339.8 ± 7.5 (t(21) = 3.95, *p* =.001, d = 1.16), and for the OUT, 527.2 ± 39 versus 445.4 ± 55.3 (t(21) = 2.57, *p* =.02, d = 0.37) condition. Hence, the patterns of saccade amplitudes and latencies were fairly consistent with Experiment 1.

## Discussion

The present study aimed at investigating the role of perceptually filled-in stimuli in the control of eye movements. We tested three viewing conditions that gave rise to veridical sensory information (binocular vision, monocular vision with target outside of blind spot) or filled-in percepts (monocular vision with “invisible” target inside of blind spot). Participants performed an orientation discrimination task with high accuracy for all viewing conditions. Importantly, however, their performance deteriorated when they did not make a saccade to the invisible target in perceptually filled-in viewing conditions. Participants’ orientation discrimination performance was best when they made a saccade to the target side given that the target was close to the fovea after the saccade. Performance decreased when the target was presented outside of the blind spot and participants made a saccade to the other side. In this case, the target was initially in the periphery at a medium eccentricity and was shifted to an even higher eccentricity as participants moved their gaze to the other side. Performance further dropped when the target was presented inside of the blind spot and participants made a saccade to the other side. In this case, the target was initially not visible and only detected in the far periphery. In Experiment 1, our results showed that eye movements were not preferentially directed at the blind spots when “invisible” targets in the left and right blind spot occurred interleaved. This suggests that observers treated the inferred information in the blind spot as veridical and reliable. In Experiment 2, we found that individuals learned to look preferentially at the blind spot, when viewing conditions were blocked, such that “invisible” targets occurred always in the same blind spot side. This indicates that eye movements could be optimized by using contextual information and reinforcement learning, in line with previous research^60,61^.

We found that eye movements do not preferentially test perceptual inferences in the blind spot in general. Previous research showed that filling-in renders natural scotomata invisible for perception and for meta-cognitive decision making^34,36^. Extending these findings, our results show that the high uncertainty at the blind spot is not taken into account even for visual search behavior. The filled-in information is treated as veridical as sensory information in intact regions. Perceptual filling-in at the blind spot occurs almost instantaneously, in early visual processing^30^ and precedes pop out in visual search^24^. Notably, humans are unaware of the information about which eye receives visual input, yet this information can be decoded from the activity in the visual cortex^62,63^. Our results complement these previous findings by showing that information about the eye-of-origin and about filling-in is not used to optimize eye movements in a visual search task.

When viewing conditions were blocked, such that the “invisible” target in the blind spot occurred always on one side, participants learned to make saccades to the blind-spot side. Importantly, this was not done at the expense of missing visible targets on the other side, which means that the participants learned to make a saccade to the blind-spot side whenever they could not perceive the target on the other side. How did they do so? One could imagine that participants automatically end-up with the correct solution if they were trying to balance the proportion of saccades to the left and right side. They would always make a correct saccade to the non-blind-spot side whenever the target appeared there and make a saccade in the other direction in the remaining 50% of trials to balance both sides. However, this was not the case at the beginning of the experiment, where they made saccades to both sides equally often whenever the target was invisible in the blind spot. This suggests that merely balancing saccades in both directions cannot explain the behavioral pattern. Alternatively, participants might have learned that whenever the target is initially invisible in the periphery, it will appear after a saccade to one particular location. Previous studies showed that finding a previously invisible target at a certain location reinforces saccades to that location^60,61^. Such a reinforcement learning process might have driven the learning in our paradigm as well.

A modification of eye movement control is also necessary in the case of pathological scotomata that can occur due to ocular diseases, such as age-related macular degeneration^64^ or due to lesions in visual cortex, such as hemianopia^65^. For example, it has been shown that in the case of artificial central vision deprivation, human subjects benefit from eye movement training that provides them with a new preferred eccentric retinal locus for fixation^66–68^. Patients with ocular diseases or visual pathway lesions process visual information differently from healthy controls, being affected in various daily activities such as grasping, reading or driving, and visual search or smooth pursuit^69–71^. Typically, these patients demonstrate longer search behavior with more saccades to the same object or more distributed fixation positions^72–74^. Similar to our monocular visual search paradigm, the challenge is to make saccades towards a part of the visual field, where information about the environment is maximally uncertain, but that also contains no salient visual information to attract eye movements. The visibility of the scotoma seems to play a crucial role. Patients who are aware of their scotoma are less impaired in reading^75^ and raising awareness improves reading performance^76^. Consistently, healthy human participants are able to adjust their eye movements to artificial scotomata that are visible^77^. When the scotoma is invisible, such as in our case of the blind spot or in the case of pathological scotomata, consistently making eye movements to the scotoma can trigger a learning process. Such training can even lead to improvements in everyday task performance of patients^78^. Our results show that there is no advantage for the blind spot as a natural scotoma compared to artificial or pathological scotomata. In all of those cases, search behavior needs to be optimized by learning processes. This is somewhat different from the scotopic foveal scotoma, which is caused by the absence of rod photoreceptors in the fovea. Under dim lighting conditions, eye movements seem to be adjusted to preserve performance in different visual tasks, such as visual search^79^ or reading and face recognition^80^.

While we found an improvement in the proportion of saccades to the blind spot side when conditions were blocked in Experiment 2, saccade latencies and amplitudes showed persistent differences between saccades to visible and invisible targets: amplitudes were smaller and less accurate for all saccades to the side without a visible target (either to a target in the blind spot or to the other side of visible target) and latencies were longer when the target was presented in the blind spot. These differences could reflect differences in eye movement control between reactive saccades that are triggered by a visible target and voluntary saccades that are used to explore the noise patch on one of the two sides. Voluntary saccades have typically longer latencies than reactive saccades^81,82^. The absence of a clear visual target and therefore larger spatial uncertainty can contribute to smaller saccade amplitudes^83,84^. In this perspective, learning may not be able to change which control mechanism is used for the saccade to improve saccade latencies and amplitudes.

Our results show that eye movements do not specifically target the location of the blind spot as a region of maximal uncertainty in monocular visual search. This indicates that the inferred information in the blind spot is not treated differently than veridical sensory information elsewhere for eye movement control. Learning processes can optimize eye movements only when the high-uncertainty blind-spot location is consistent and predictable across multiple trials. These findings could be used to optimize oculomotor rehabilitation in patients with pathological scotomata.

## Data Availability

The data supporting the findings of this study and the statistical analysis code used in the manuscript are available at https://doi.org/10.5281/zenodo.17669770. Further information and source requests should be directed to the corresponding author, Cemre Baykan, Philipps-Universität Marburg, Fachbereich Psychologie, AG Sensomotorisches Lernen, Marburg, Hessen, Germany, or via e-mail: baykan@staff.uni-marburg.de.
